# TiNi-Based Material with Shape-Memory Effect for Surgical Treatment of Diseases of Small Intestine in Newborn and Young Children

**DOI:** 10.3390/jfb14030155

**Published:** 2023-03-14

**Authors:** Sergey G. Anikeev, Maria I. Kaftaranova, Valentina N. Hodorenko, Stanislav D. Ivanov, Nadezhda V. Artyukhova, Anastasiia V. Shabalina, Sergei A. Kulinich, Grigory V. Slizovsky, Anatolii V. Mokshin, Victor E. Gunther

**Affiliations:** 1 Laboratory of Medical Materials Science, Tomsk State University, 634050 Tomsk, Russia; 2 Institute of Physics, Kazan Federal University, 420008 Kazan, Russia; 3 Department of Pediatric Surgical Diseases, Siberian State Medical University, 634050 Tomsk, Russia; 4 Research Institute of Science and Technology, Tokai University, Hiratsuka 259-1292, Kanagawa, Japan; skulinich@tokai-u.jp

**Keywords:** TiNi, structure, martensitic transformations, strength, double-barreled enterostomy, compression anastomosis

## Abstract

Alloys based on TiNi are widely used in various fields of technology and medicine. In the present work, we report on the preparation of TiNi-alloy-based wire with the shape-memory effect, which was used for compression clips for surgery. The composition and structure of the wire and its martensitic and physical–chemical properties were studied using SEM, TEM, optic microscopy, profilometry, mechanical tests, etc. The TiNi alloy was found to consist of B2 and B19′ and secondary-phase particles of Ti_2_Ni, TiNi_3_ and Ti_3_Ni_4_. Its matrix was slightly enriched in Ni (50.3 at.% of Ni). A homogeneous grain structure was revealed (an average grain size of 19 ± 0.3 μm) with equal quantities of grain boundaries of special and general types. The surface oxide layer provides improved biocompatibility and promotes the adhesion of protein molecules. Overall, the obtained TiNi wire was concluded to exhibit martensitic, physical and mechanical properties suitable for its use as an implant material. The wire was then used for manufacturing compression clips with the shape-memory effect and applied in surgery. The medical experiment that involved 46 children demonstrated that the use of such clips in children with double-barreled enterostomies permitted improvement in the results of surgical treatment.

## 1. Introduction

Alloys based on TiNi are widely used in various fields of technology and medicine. This is due to their unique functional properties, shape-memory effects and super elasticity, as well as a high level of biocompatibility [1,2,3,4,5,6,7,8,9,10,11,12,13]. The latter is ensured by their corrosion resistivity and electrochemical passivity of the superficial oxide layer composed of TiO_2_ [14,15,16,17,18,19,20,21,22,23]. Moreover, the physical–mechanical characteristics of such alloys are close to the characteristics of body tissues [24,25,26,27]. More specifically, the hysteretic type of behavior, the correspondence of damping properties and wettability of the material to biological tissues, and the reliability of functioning in the body under conditions of alternating exposure make it possible to use TiNi-based alloys in various fields of medicine to solve different complex problems. In particular, TiNi-based implants are used in traumatology, reconstructive surgery, oncosurgery, surgical treatment of urological and gynecological diseases, vascular surgery, as working elements for orthodontic appliances, devices in ophthalmology, etc. [28,29,30,31,32,33].

The use of TiNi shape-memory implants in gastrointestinal surgery was described in [34,35,36,37]. The development and creation of TiNi-based implants for the formation of anastomose initiated a new direction in the improvement of the compression suture [38]. Thus, fundamentally new possibilities appeared in surgery that permit modeling seamless anastomoses of required configuration between the hollow organs of the gastrointestinal tract.

The mechanism of compression suture is based on the optimal, from the view point of tissue trophism, compression when the walls of connected organs come into contact [38,39]. One of the main advantages of compression anastomosis is the absence of foreign bodies along the suture line. This leads to a decrease in inflammation in the healing zone. In addition, a compression suture, unlike a manual suture, is devoid of such disadvantages as the formation of coarse scar tissue and contamination of the suture channel [38,40,41].

The use of magnets [40,42,43] and TiNi shape-memory alloys [8,39,41,44,45] were described for the formation of compression sutures in children. For the first time, devices made of TiNi and used for compression anastomoses were developed and widely used in 1985 under the guidance of Professor R.V. Ziganshin [36].

When cold, TiNi-based materials can be given the necessary shape which is then restored upon subsequent heating [24]. Hence, the effect of uniform compression on tissues resisting the restoration of the original shape made it possible to develop new techniques and methods for modeling seamless connections between hollow organs [38].

A large number of studies on the use of TiNi alloys in abdominal surgery in adults were performed in Tomsk under the guidance of V.E. Gunther, G.T. Dambaev and others. The number of such works in pediatric abdominal surgery is limited.

This work studied a material based on the TiNi alloy in the form of wire that was used in medical practice as clips for the treatment of diseases and injuries of the small intestine in newborns and young children (up to one-year-old). A detailed medical investigation was provided in our previous work [45]. Here, we reveal the structure and properties of the used TiNi-based material with shape-memory effect and analyze, in this regard, its effectiveness in the formation of delayed compression anastomosis in a double-barreled Mikulicz enterostomy in young children. 

For the first time, here we report on the clinical use of TiNi-based shape-memory devices applied to treat the formation of compression anastomosis in the area of double-barreled enterostomes in newborns and young children, including premature babies. First, we describe the structural features and physical and mechanical properties of the TiNi-based material used to manufacture such compression clips, after which their medical application is demonstrated. The novelty and originality of the work thus lie in the successful introduction into medical practice of TiNi-based devices for forming double-barreled enterostomas, as well as in the use of comparative analysis with the results of children treated with double-barreled enterostomies without forming a compression anastomosis.

## 2. Materials and Methods

The monolithic alloy based on TiNi studied in this work was obtained by remelting plates of nickel (N1, purity of 99.90%, “Ural Metals”, Kamensk-Uralsky, Russia) and spongy titanium (TG-90, purity of 99.94%, “Ural Metals”, Kamensk-Uralsky, Russia) in an ISV-0.004-PI M1 (Petra, Ufa, Russia) induction furnace filled with inert argon gas (99.99% “Cryogenmash-Gas”, Tomsk, Russia). The initial components were weighed in an equiatomic ratio using a GH-200 balance (A&D, Tokyo, Japan). The resulting cylindrical ingots (300 mm long and 25 mm in diameter) were sequentially processed in a DOU-80 rolling mill (DOU, Moscow, Russia), an RKM-4 rotary forging machine (Pressmash, Taganrog, Russia), and an SVP-0.12M drawing machine (Pressmash, Taganrog, Russia) at intermediate annealing temperatures of 450–950 °C in tube furnace SUOL 0.4.4/12 (Tula-Term, Tula, Russia). The final product diameter achieved was 1 mm. To study the structure and properties of the resultant TiNi alloy, samples with lengths of 10–40 mm were cut from the obtained wire using an electroerosive machine ARTA 123 PRO (“NPK “Delta-Test”, Fryazino, Russia).

To study the macro- and microstructure of the alloy, its metallographic sections were prepared in accordance with commonly accepted protocols using sandpaper of different grades [46,47]. At the final stage, the sample was polished using a diamond paste (1–3 µm, Struers, Cleveland, OH, USA). After obtaining a thin section, the sample was washed in alcohol (96%, extra pure, HIMMED, Moscow, Russia) and dried on filter paper (Struers, Cleveland, OH, USA). During grinding and polishing, the surface of the material was monitored using an Axiovert-40MAT microscope (Carl Zeiss, Oberkochen, Germany).

To reveal the material’s grain microstructure, etching was performed, for which the material was immersed in acidic solution (3H_2_O + 2HNO_3_ + 1HF). Nitric (65%) and hydrofluoric (45%) acids, both chemically pure, were purchased from SIGMATEK (Khimki, Russia). After that, the material was rinsed with water and alcohol. The type of grain boundaries was determined using the method of different boundary etchability. The classification of grain boundaries according to the general and special types was carried out according to the degree of their etching. The average grain size was determined by means of the method of random cross-sections in accordance with the standard procedure previously reported elsewhere [48].

The microstructure of the alloy was studied using a Philips SEM 515 (Philips, Amsterdam, The Netherlands), Quanta 200 3D (FEI, Hillsboro, OR, USA) scanning electron microscopes and transmission electron microscope Hitachi HT-7700 equipped with a scanning mode unit and a Bruker X-Flash 6 T/60 V energy-dispersive spectrometer (Billerica, MA, USA). For TEM, cross-sectional samples were prepared from pore walls using a Hitachi FB-2100 focused ion beam (FIB) system. The elemental composition was studied using an EDAX ECON IV microanalyzer (EDAX, Mahwah, NJ, USA). The phase composition of the alloys was studied by XRD on an XRD 6000 diffractometer (Shimadzu, Kyoto, Japan). The patterns were recorded within the 2Θ range of 30–130° using CuK-radiation, after which the PDF-4 database and Powder Cell 2.5 software with pseudo-Voigt profile function were used for phase analysis. Three-dimensional reconstruction of the sample surface and evaluation of its roughness parameters were performed using an optical profilometer MNP-1 (TDI SIE SB RAS, Novosibirsk, Russia).

The characteristic MT temperatures were estimated by measuring temperature dependences of electrical resistance using a SIES-30 setup (Kristallooptika, Tomsk, Russia). The main fracture characteristics, such as tensile strength $\left(\sigma_{f}\right)$ and strain to failure $\left(\varepsilon_{f}\right)$ of the alloy, were recorded from the curves *σ*(*ε*) obtained on an Instron 3369 setup (Instron, Norwood, MA, USA).

The tensile strength of the samples was determined as follows [24,25]:

(1) $$\sigma =\frac{P}{S},$$

where *P* is the load applied to the sample; and *S* is the cross-sectional area of the sample.

The strain to failure of the alloy was determined by the formula:

(2) $$\varepsilon =\frac{l-l_{0}}{l_{0}}\times 100\%,$$

where *l*_0_ is the initial length of the sample; *l* is the length of the deformed sample. 

The main MT characteristics are *M_s_* and *M_d_* temperatures and minimal and maximal martensitic transformation stresses $\tau_{max}^{M_{d}}$ and $\tau_{max}^{M_{d}}$. Their values were determined using the temperature dependence σ(T). The numerical values of $\tau_{max}^{M_{d}}$ and $\tau_{max}^{M_{d}}$ were found from the stress at the corresponding points of *M_s_* and *M_d_*. The temperature *M_d_* (the maximal temperature of the formation of martensite under stress) was determined as the maximum in the temperature dependence σ(T). To study the temperature dependence of the critical martensitic transformation stresses in a wide temperature range, at the first stage, samples were deformed below the temperature range of MTs. Then, without unloading, the samples were heated while simultaneously measuring the level of stress that the sample developed when attempting to restore its original shape [24]. 

A clinical study on the use of double-barreled enterostomy with compression anastomosis was conducted at the Department of Pediatric Surgical Diseases of the Siberian State Medical University and approved by the local ethical committee (protocol No. 7936 of 28 October 2019). The Regional Perinatal Center (named after I.D. Yevtushenko) and the Emergency Hospital No. 2, Tomsk, were the base of the study. 

The study included 46 children under the age of 1 year who were operated on in the above-mentioned hospitals for the period from 2000 to 2021 for congenital and acquired pathology of the jejunum and ileum of the small intestine.

A double-barreled enterostomy, according to Mikulich, was applied by matching and parallel orientation of the afferent and efferent ends of the small intestine and fixation with interrupted sutures for 2.5–3 cm along the antimesenteric edge, with the removal through a separate incision or laparotomic wound with layer-by-layer fixation in the abdominal wall. The imposition of a compression TiNi clip (with shape-memory effect) on the interstoma spur of double-barreled enterostomas was carried out in the experimental group at various times (9–58 days) of the postoperative period.

In the experimental group, 18 children aged from 1 day to 6 months were operated on with the imposition of a double-barreled enterostomy and subsequent formation of a compression anastomosis. According to the sex ratio, there were 11 boys and 7 girls. The average gestational age was 35 (27–39) weeks. The average body weight at birth was 2025 (750–3192) g. There were 10 premature babies and 6 with extremely low body weight. Enterostomies were placed on day 20 of life on average (from day 1 to day 80). The average body weight at enterostomy was 2676 (1343–3390) g.

The clip was applied to the interstoma spur 14–46 days (23 days on average) after enterostomy. Indications for the use of a compression device were pathological loss of chyme (more than 20 mL × kg per day) in the presence of contraindications to the closure of the stoma. The average body weight, when applying the clip, was 3074 (2421–3900) g, the minimum being 1220 g. The clip was removed on its own on day 5 on average (from day 3 to day 7). The feces were found to pass through the anus on their own from day 1 to day 3 after the removal of the clips.

**Figure 1:**
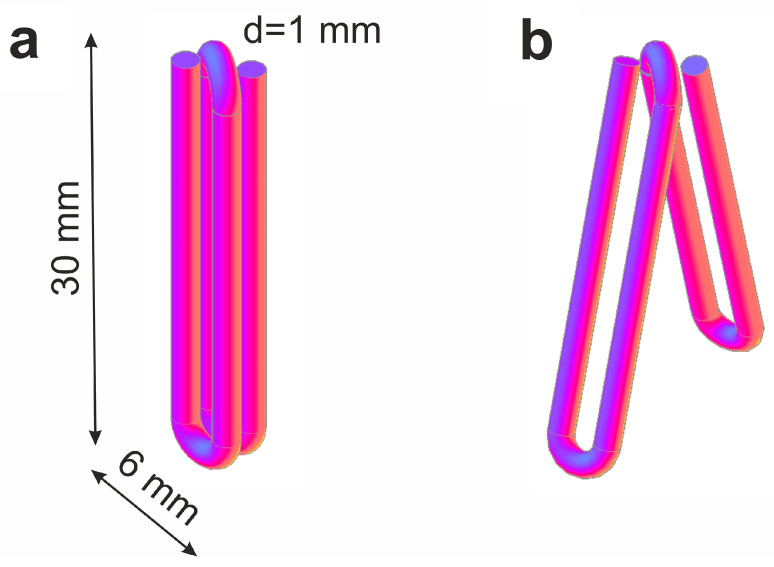
Model of compression clip made of TiNi with shape-memory effect with its closed (**a**) and open (**b**) branches.

As shown in Figure 1, the used clips were made of TiNi alloy and consisted of two parallel-oriented turns-branches 30 mm × 6 mm in size each. The wire cross-section diameter was 1 mm. The clips were pre-cooled at 0 °C and then, in their open state, were applied on an interstoma spur. When such a clip is warmed up to the body temperature, its branches should close, and the compression of the adjacent walls of the enterostomy inlet and outlet sections should appear. As a result of the adhesion-necrotizing process, an inter-intestinal anastomosis is formed, and patency to the intestines distal to the stoma is restored.

The study analyzed: gender, gestational age and birth weight; age and body weight at the time of imposition and closure of the stoma; complications associated with the formation and closure of stomas and their assessment according to the Clavien–Dindo Classification (CDC) of surgical complications [49].

Data processing was carried out using the software package for statistical analysis IBM SPSS Statistics v.23 on the operating system Windows v.11. For non-normally distributed quantitative data, the significance of differences was assessed using the nonparametric Mann–Whitney U-test for two independent groups. To assess the significance of differences in qualitative data in groups, the χ^2^-Pearson test for two independent groups was used, and at a frequency of less than 5, Fisher’s exact F-test was used. Differences were considered statistically significant at *p*-value < 0.05.

## 3. Results

### 3.1. Macro- and Microstructural Features of TiNi Wire Material

The procedure of obtaining and thermo-mechanically processing the material is known to control the structural features of TiNi alloy. The latter features determine the material’s behavior during martensitic transformations (MTs). In TiNi, MTs are accompanied by the formation of martensite plates and their subsequent growth. Therefore, in our experiments, the composition of the TiNi phase and the mode of heat treatment during the preparation of wire blanks determined their characteristic MT temperatures and the sequence of phase transformations [24,25]. The size and distribution density of precipitated particles in the matrix also have a significant effect on the behavior of both the characteristic MTs and the physical–mechanical properties of such alloys [24,25,50,51]. Hence, the martensitic features of the material are determined not only by its TiNi phase composition but also by the size and shape of secondary particles, as well as their density and distribution. 

Grain boundaries are also known to have a significant effect on the nucleation and growth of martensite crystals. Such boundaries can act as sites of predominant nucleation of martensite crystals and, consequently, can control the MT temperature. At the same time, they can act as stoppers preventing the development of MT [51]. The grain size and the ratio of grain boundary types (general and special types) affect both the conditions for the nucleation of martensite crystals and the nature of the MT in a polycrystalline ensemble [50]. When the grain size decreases, the bulk density of the boundaries increases. At this point, conditions for the nucleation of martensite crystals and the nature of MT propagation in a polycrystalline ensemble change [24,25].

Thus, detailed studies on the TiNi alloy structure and, in particular, its secondary particles and grain boundaries are an important part of the development of new implant materials. 

#### 3.1.1. Secondary Phase Inclusions

**Figure 2:**
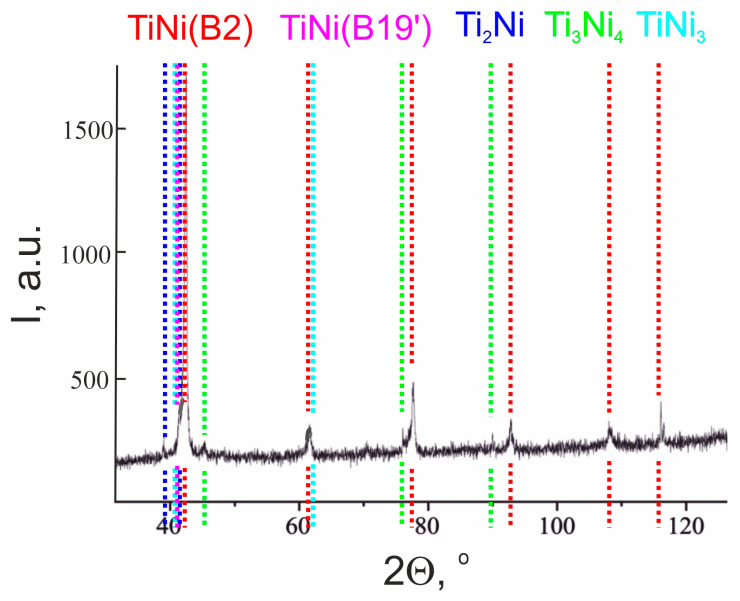
XRD pattern of monolithic TiNi wire.

Previous studies on TiNi alloys showed that microstructurally, along with the TiNi matrix phase available in two states (B2 and B19′), the materials have secondary-phase particles of Ti_2_Ni, TiNi_3_ and Ti_3_Ni_4_ (Figure 2). The calculated content and lattice parameters of all the phases are presented in Table 1, where a phase composition typical for TiNi-based monolithic alloys obtained by induction melting followed by drawing is shown.

**Figure 3:**
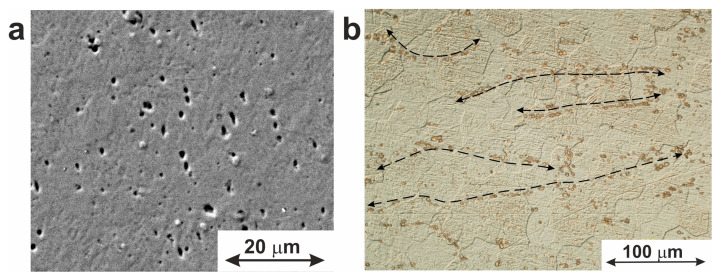
Microstructure of TiNi wire with Ti_2_Ni particles and oxycarbonitrides based on the (**a**) SEM image; (**b**) optical microscopy image showing particle distribution along the longitudinal drawing direction.

Figure 3 shows microscopic images of the material used in this work, with both the TiNi matrix and intermetallic precipitates with sizes from 0.1 to 5.5 µm being well seen. The latter particles are seen to be enriched in titanium and are located both inside the grains and along their boundaries. A characteristic feature of such particles is their geometric shape which varies from round to pyramidal. The Ti:Ni ratio in the detected particles was found to be 2:1, thus implying the stoichiometric Ti_2_Ni phase as their main component. It is known that during the drawing process, plastic deformation of material occurs, and brittle particles of Ti_2_Ni phases (and/or oxycarbonitrides based on them) are destroyed. This leads to the appearance of the “lines” containing Ti-enriched particles in the longitudinal direction of the wire. Such particles are well-known for their incoherent conjugation with the TiNi matrix [24]. According to the phase diagram of the Ti–Ni system, the Ti_2_Ni phase is formed during crystallization via peritectic reaction [26]. Segregation of a large number of large Ti_2_Ni particles within the TiNi (B2) matrix can lead to changes in the chemical composition of the TiNi matrix so that its enrichment in nickel up to 50.3 at.% can be reached.

**Figure 4:**
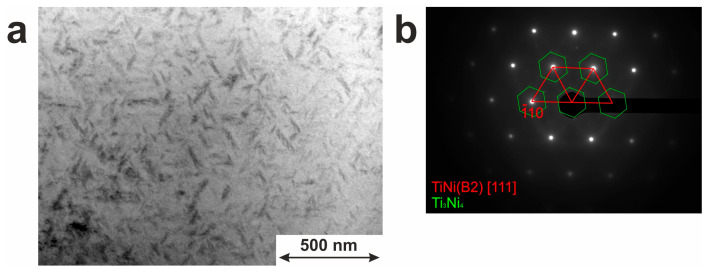
Microstructure of TiNi wire with Ti_3_Ni_4_. (**a**) Bright field image; (**b**) SAED pattern.

TEM revealed finely dispersed coherent particles with sizes from 10 nm to 150 nm, as well seen in Figure 4a. The average particle size was 81 nm with a standard deviation of 30 nm. SAED pattern showed the presence of Ti_3_Ni_4_ inclusions (Figure 4b). Due to their small size and coherent coupling with the TiNi matrix, such Ti_3_Ni_4_ particles are expected to contribute significantly to the behavior of the characteristic temperatures and parameters of shape-memory effects in this material.

The precipitated dispersed particles are known to control the processes of nucleation and growth of martensitic crystals [50]. They can act as obstacles for the movement of the interfacial boundary or can be the preferential sites for the nucleation of B19′ or R martensite crystals [51]. In the present work, fine Ti_3_Ni_4_ particles are not localized along grain boundaries or bodies but are seen in Figure 4a to be uniformly distributed within the TiNi matrix. 

**Figure 5:**
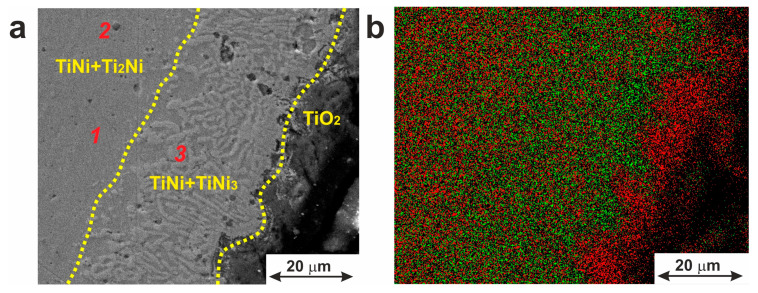
(**a**) Microstructure of a cross-section of the surface and subsurface layers of TiNi wire. Red numbers indicate three positions of EDX analysis with the results presented in Table 2. (**b**) Elemental distribution map: red for Ti and green for Ni.

The presence of the TiNi_3_ compound at the periphery of the wire was confirmed by cross-sectional SEM (see Figure 5a). The TiNi_3_ inclusions are seen in Figure 5a to have a dendritic morphology, with the thickness of the TiNi + TiNi_3_ layer being around 24–27 µm. 

During the wire drawing, intermediate annealing is performed to relieve stresses that have arisen after its plastic deformation. Annealing of a TiNi wire in the air is accompanied by oxidative processes during which the following reaction occurs: TiNi + O_2_ → TiNi_3_ + TiO_2_. The results obtained in our work are consistent with the work of Zhu and coworkers [52]. Moreover, the authors of this work mentioned that under extreme conditions, at temperatures as high as up to 1000 °C, or during long-term annealing (up to 300 min), the formation of pure Ni, as well as diffusion porosity under the oxide layer, could be observed [52]. Such treatments were excluded in the present study, which could be the reason why neither pure Ni nor porous sublayer under the surface oxide layer was found in our material.

Thus, the TiNi wire used in this study consisted of B2 and B19′ phases with inclusions of secondary-phase particles of Ti_2_Ni, TiNi_3_ and Ti_3_Ni_4_. Additionally, chemical analysis revealed that its TiNi matrix was slightly enriched in Ni (50.3 at.% of Ni). This was expected to ensure the implementation of the shape-memory effect. Moreover, the Ti_3_Ni_4_ inclusions were shown to retain coherent conjugation with the TiNi matrix, probably due to their sizes of around 81 nm. This would also affect the martensitic behavior of the material.

#### 3.1.2. Superficial Layers

The state of the surface layer of material aimed for medical application is of special importance. During preparation processes, such a layer delivers lubricants to the plastic deformation zone of the dies [53]. In addition, the presence of such a granular layer prevents the TiNi wire material from sticking to the working surfaces of the dies during thinning. This further reduces the friction coefficient at different degrees of deformation. A combination of various thermomechanical treatments (such as lubricants, adhesive coatings for lubricants, and high-strength coatings used on the working cone of the die) allows one to reduce the drawing force and the number of wire breaks, thus varying the quality of the product’s surface layer [53].

The distribution of chemical elements within the material’s cross-section can be seen as an EDX map in Figure 5b. Analysis of this map reveals that the surface is composed of a TiO_2_ layer (12.8 ± 2.6 μm) and two separated TiNi layers with inclusions of phases TiNi_3_ and Ti_2_Ni. Table 2 shows the chemical composition of the phases according to EDX data. No pure Ni was found under the oxide layer or on its surface. The presence of some amount of carbon (not shown here) is explained by the use of hydrocarbon lubricants in the process of the wire material’s drawing. 

**Figure 6:**
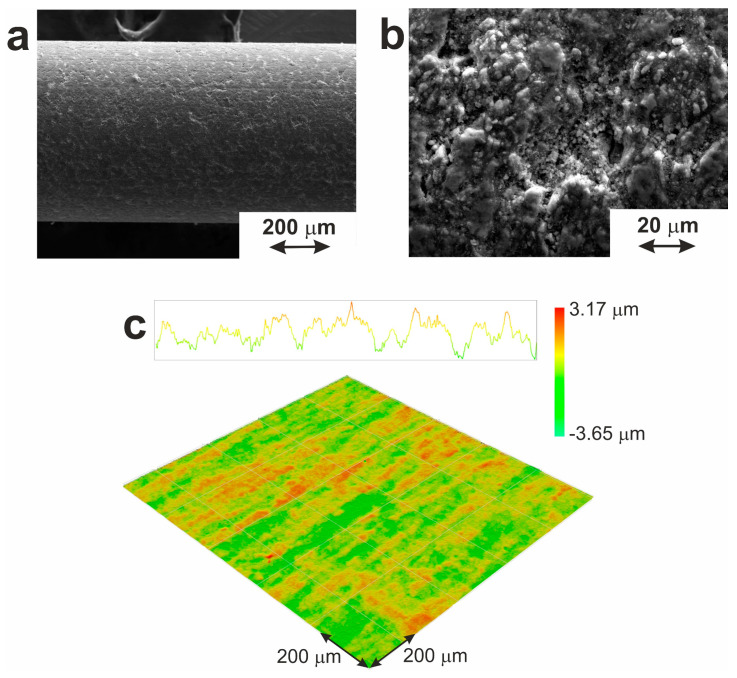
Surface morphology of TiNi wire according to (**a**,**b**) SEM, and (**c**) optical profilometry.

As seen in Figure 6c, optical profilometry showed that the surface oxide layer had a developed granular structure with longitudinal and transverse cavities formed during the drawing process. The average deviation of the surface profile (R_a_) and the height of the profile irregularities (R_z_) were found to be 0.5 and 5.5 µm, respectively. 

The response of biological tissues to the implant is known to be determined by the morphology and phase, and chemical composition of its surface [54]. Taking titanium alloys as the standard for implant material [55], different information about the optimal surface roughness can be found [56]. This is explained by different tools used to determine roughness parameters (atomic force microscopy, contact and optical profilometry), different types of materials and different areas evaluated, as well as a variety of biological tissues (hard and soft ones) whose reaction was studied during interaction with the implant. The results of the present work are in good agreement with the work of Ungersbijck and co-authors, who explored the interaction of soft tissues with various materials based on titanium and stainless steel [57]. They showed the best adhesion of a thin, soft tissue layer to the implant of anodized titanium with a coarse surface (R_a_ = 0.78 ± 0.1 µm and R_z_ = 6.65 ± 1.2 µm).

Thus, the resulting material has a composite structure based on a metal matrix and surface oxide layer. The latter surface layer with a developed granular structure is formed during the drawing process, and its presence provides increased biocompatibility, promoting the adhesion of protein molecules on the surface [53,58,59,60]. Moreover, the oxide layer on its surface has a thickness that is far enough to prevent the release of Ni ions at a harmful level into surrounding tissues. 

#### 3.1.3. Grain Boundaries

It was mentioned above that the grain boundaries can affect the nucleation and growth of martensite crystals, controlling the MT temperatures [61,62,63,64,65,66,67,68,69,70,71]. A decrease in grain size, with a subsequent increase in the bulk density of the boundaries, is known to affect the nucleation of martensite crystals and the nature of MT propagation in a polycrystalline system [72,73]. In the present work, the average grain size was found to be 19 ± 0.3 µm, implying that the grains shrank somehow in comparison with the initial material used for the wire preparation (25–28 µm).

Importantly, the grain boundaries are assigned to two different types, general and special types. Changes in the properties of the boundaries during heating can lead to changes in the strength and plasticity of materials and affect the course of recrystallization processes [74,75]. Visual identification of special boundaries in the structure of polycrystals is based on their special features, for instance, low etchability [75,76,77,78]. The boundaries of the general type have higher energy than the former type since they are more strongly etched in most electrolytes [75,76,77,78]. Thus, in the present study, the type of boundaries was determined by assessing the degree of etching. The fraction of special-type boundaries detected in the studied material was 52.6%. 

**Figure 7:**
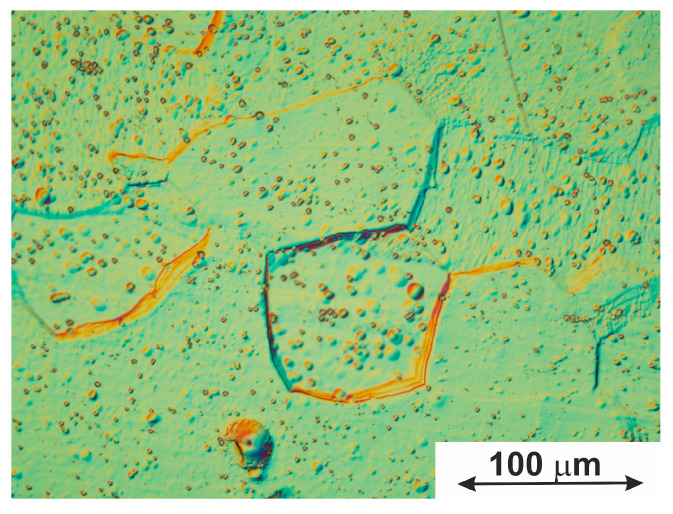
Optical microscopy image of metallographic sample demonstrating the general-type grain boundary migration.

For the material studied in this work, the processes of migration of boundaries of a general type were detected in some local areas. As a result, a group of boundaries of a special type was formed in those areas, as seen in Figure 7. Thus, the formation of special grain boundaries is determined by the migration ability of general-type boundaries and the intensity of recrystallization processes during the thermomechanical processing associated with drawing (i.e., when the specimen was formed). In turn, it is known that the migration ability of general-type boundaries in multiphase alloys depends on the presence of secondary phases at the grain boundaries [74,76]. In addition, in this sense, the boundaries of the special type are almost always free of segregated particles.

Thus, the material obtained has a homogeneous grain structure with an average grain size of 19 ± 0.3 μm, and a ratio of grain boundaries of special and general types was found to be close to 1. The latter fact should have a positive effect on the development of MTs in a TiNi-based polycrystalline system since special grain boundaries do not act as barriers that slow down MT processes. This increases the shape change resources, which is important for a TiNi-based shape-memory alloy that is used as an implant material.

### 3.2. MTs and Shape-Memory Effects

In terms of behavior in the human body, the optimal implant should be similar to living tissue. Namely, it should have a given hysteresis on the load–unload deformation diagram. In addition, the degree and magnitude of shape recovery should correspond to the required value and degree of tissue shape recovery [24,25].

**Figure 8:**
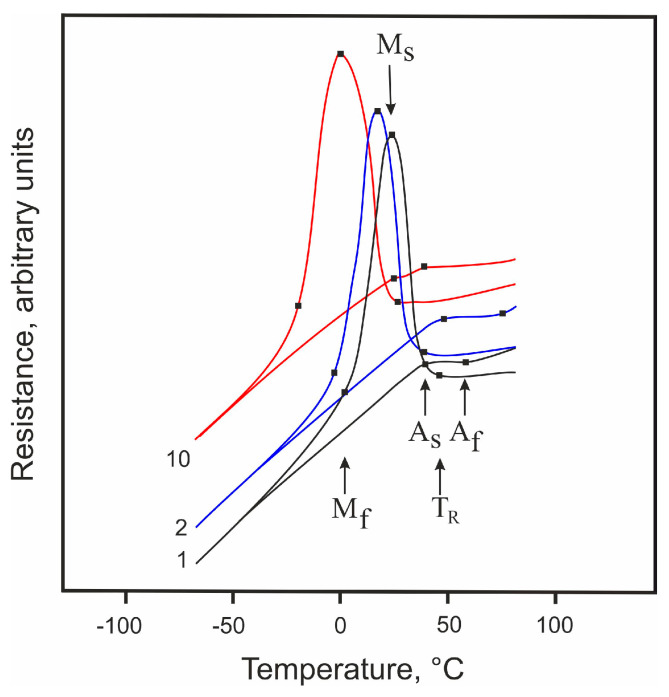
Temperature dependence of the electrical resistivity of TiNi wire as observed for 1, 2, and 10 cycles.

Analysis of the temperature dependences of the electrical resistance for the studied material showed that its MTs occurred in two stages, i.e., as: B2 🡪 R 🡪 B19′ (Figure 8). It can be confirmed by the characteristic rise in electrical resistance values observed at a temperature of T_R_ = 44 °C [79]. Moreover, in the presence of fine Ti_3_Ni_4_ particles detected in the studied material (see Section 3.1), this type of phase transition (through the R-phase) is favorable. The Ti_3_Ni_4_ particles can be nucleation sites for R-phase crystals since the lattice distortion associated with the R-phase transformation (about 1%) is much smaller than that with the B19′ transformation (about 10%).

Thermal cycling is known to lead to a shift in MT temperatures to the low-temperature region. Graphically, only direct MT temperatures can be reliably determined from curves such as those shown in Figure 8. For the 10th cycle, the shift of T_R_, M_s_, and M_f_ temperatures is seen to reach 20 °C (Table 3). The reason for this shift observed during thermal cycling can be phase hardening, the phenomenon that consists in the accumulation of dislocations during MTs [80]. During the direct MT with large hysteresis, martensite crystals with dislocations, twins, and stacking faults are formed, and after the reverse transformation, the initial phase still contains a high density of dislocations [81,82]. Moreover, the process of complete MT is accompanied by inevitable damage to the interfacial boundaries of the martensite phase [83,84,85,86,87,88]. This is a specific feature of the complete MT during the interaction of crystals of the martensite phase. 

Thus, in accordance with Figure 8, a two-stage MT takes place in the TiNi wire considered in this work, with the intermediate R-phase that is formed easily because of fine Ti_3_Ni_4_ particles present in the material’s matrix.

### 3.3. Physical–Mechanical Properties

**Figure 9:**
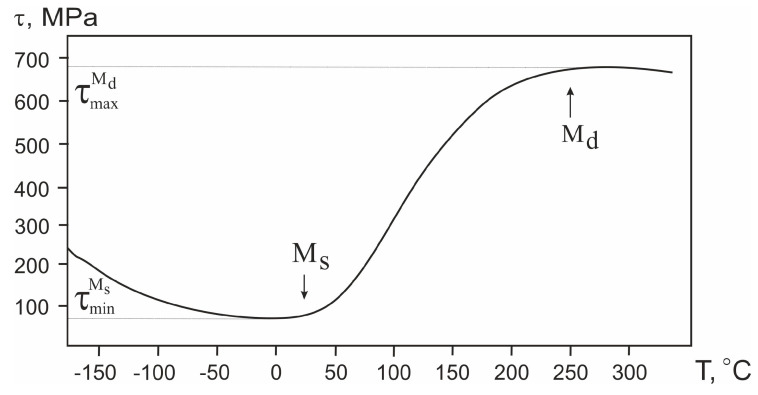
Temperature dependence of the martensitic transformation stress of TiNi wire.

The composition of the alloy (i.e., availability of additives and/or second-phase particles) determines all possible properties it will demonstrate. When it comes to shape-memory alloys, temperature becomes another extremely important experimental parameter that governs the result. In the temperature dependence of the critical martensitic transformation stresses τ(T) presented in Figure 9, the temperature range of deformation and the degree of deformation are important indicators of the material’s properties. 

In the practical use of TiNi, the difference between the maximum and minimum martensitic transformation stresses, $\tau_{max}^{M_{d}}-\tau_{min}^{M_{s}}\;$, is quite an important parameter. It determines the degree of recovery expected from the material during its behavior as a shape-memory alloy. As the difference in $\tau_{max}^{M_{d}}-\tau_{min}^{M_{s}}$ increases, the martensitic component makes the main contribution to the material’s deformation. The appearance of a plastic component of deformation is unlikely in alloys up to their temperature *M_d_*. Its contribution to the total deformation can only be observed near the *M_d_* point. According to the literature [24,25], Ti_51.15_Ni_48.85_ and Ti_50.65_Ni_49.35_ alloys with shape-memory effects have their minimum values of $\tau_{max}^{M_{d}}-\tau_{min}^{M_{s}}$ around 80 MPa and 405 MPa, respectively. In addition, for these alloys, rapid achievement of the yield strength would cause plastic deformation of the material. This causes a decrease in the value of the total accumulated deformation of the alloys. 

The maximum martensitic transformation stress $\tau_{max}^{M_{d}}$ observed for the our Ti_49.7_Ni_50.3_ alloy in the present work has a rather high value of about 680 MPa. At the same time, its minimum value of the martensitic transformation stress $\tau_{min}^{M_{s}}$ is about 70 MPa. Hence, in the present work, the $\tau_{max}^{M_{d}}-\tau_{min}^{M_{s}}$ was found to be 610 MPa. An increase in the $\tau_{max}^{M_{d}}-\tau_{min}^{M_{s}}$ (compared with the alloys described above) indicates that the deformation of the alloy is, to a great extent, due to the implementation of MT in the material rather than its plastic shear. In terms of mechanical properties, the TiNi wire with a low martensitic transformation stress of about 70 MPa is quite close to biological tissues.

Reducing the martensitic transformation stress in the temperature range characteristic of the operating conditions in the patient’s body could increase the elasticity of medical implants made of a monolithic alloy. Moreover, this can facilitate the modeling of implants for the configuration of replaced defects. The lower the martensitic transformation stress $\tau_{min}^{M_{s}}$ is, the more flexible implant made from such material will be. Thus, from the point of view of reconstructive surgery, for modeling bulky and complex implants, such material can be more accurately adapted to the replaced defect. 

We assume that the main role in the MT characteristics of the alloy studied in this work is played by its structural features. On the one hand, segregation of fine Ti_3_Ni_4_ particles is believed to lead to additional internal stresses in the material. This should stimulate the development of MT with a lower $\tau_{min}^{M_{s}}$ value. On the other hand, the presence of coherent particles finely dispersed in the alloy structure is expected to result in the strengthening of the TiNi phase, also increasing its yield strength. The formation of large Ti_2_Ni particles does not directly affect the properties of the material. However, their precipitation in the TiNi phase leads to changes in the chemical composition of the matrix phase and its enrichment in nickel.

**Figure 10:**
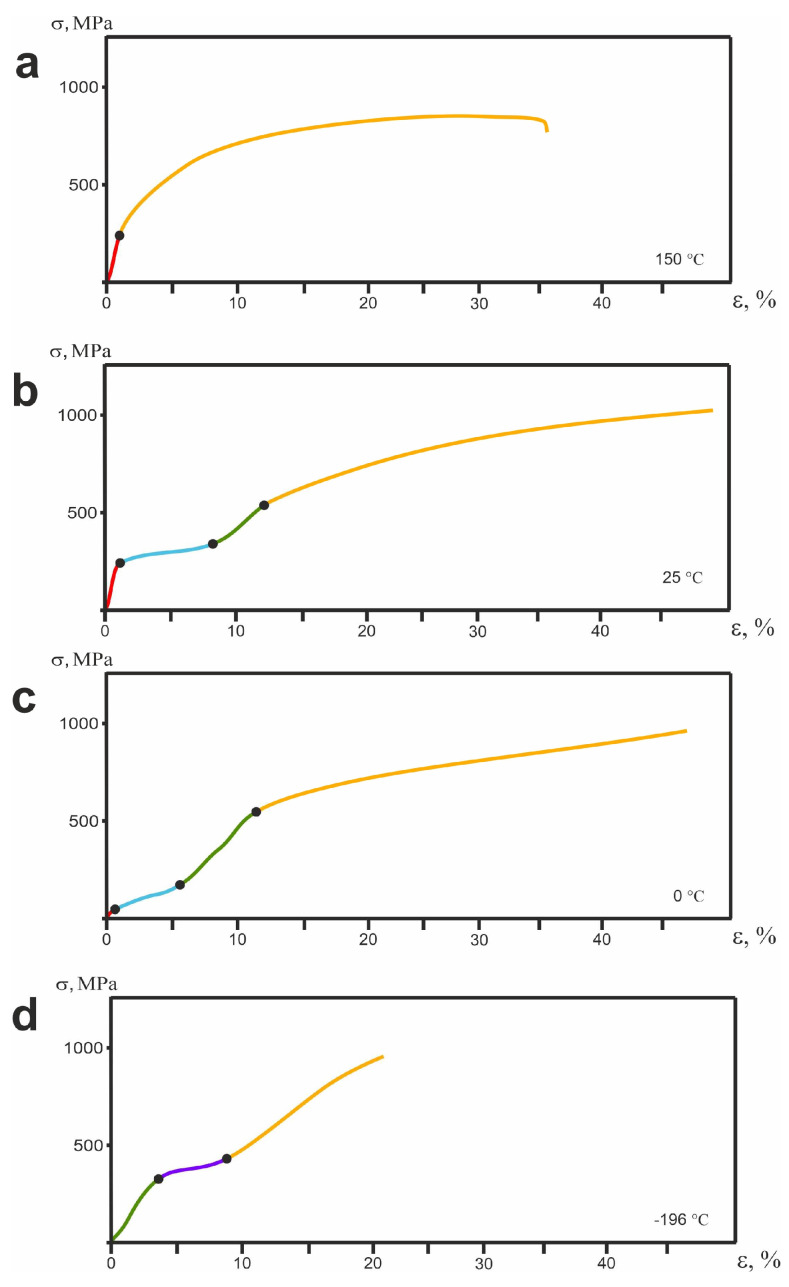
Dependence of σ(ε) for TiNi wire at different temperatures: (**a**) 150 °C; (**b**) 25 °C; (**c**) 0 °C; (**d**) −196 °C.

Figure 10 shows the stress–strain dependences σ(ε) before the failure of the alloy sample at different deformation temperatures.

Each of the deformation curves can be divided into stages characterized by different deformation mechanisms. The number of stages was different for different testing temperatures, which is explained by the different phase states of the material under testing conditions. The initial state of the material at 0 °C and −196 °C was martensitic; at 150 °C it was austenitic, and at 25 °C it was pre-martensitic.

For the initial martensitic state (at −196 °C, Figure 10d), three stages can be distinguished in the σ(ε) curve: the stage of elastic deformation of martensite (green section of the curve in panel (d)), the stage of twinning (purple section in Figure 10d), and the stage of plastic deformation (orange section in Figure 10d). For the TiNi alloy at a test temperature of 25 °C (panel (b)), the deformation is seen to begin at the stage of elastic deformation of austenite (red section in Figure 10b), then it is replaced by the stage associated with the martensitic transition (blue section in Figure 10b). The next rise of the curve is characterized by elastic deformation of the martensite (green section in Figure 10b). The final stage of this dependence is the stage of plastic deformation (orange section in Figure 10b). At 150 °C (Figure 10a), the alloy is in the austenitic state, and its dependence σ(ε) visually differs from the others and is represented by only two stages: the elastic deformation of austenite and its plastic deformation (red and orange sections in Figure 10a).

At different test temperatures, the strain curves have different stress ($\sigma_{f}$) and strain ($\varepsilon_{f}$) failure characteristics (Table 4). They are determined graphically by the breaking point seen in dependence σ(ε) curves [89]. Such a difference in the values of $\sigma_{f}$ and $\varepsilon_{f}$ of the fracture strain observed under different conditions indicates different contributions of the elastic, martensitic, and plastic components of the deformation [24,25].

At 25 °C, when the material is in its pre-martensitic state, load application was found to cause the maximum plastic deformation of the alloy at a sufficiently high level of fracture stress of −1013 MPa (Table 4). Martensite arising under the load relaxes the peak stresses in the B2 phase by martensitic reaction and plastic shear. This increases the deformation capabilities of the alloy under test conditions.

A decrease in temperature to −196 °C is seen in Table 4 to lead to a decrease in plastic properties, although the level of fracture stress is quite high (972 MPa). At this temperature, the alloy is in its fully martensitic state. Martensite has lower plastic properties than the initial B2 phase. That is why alloy deformation in this state also corresponds to a lower level of plasticity.

According to Figure 10 and Table 4, plastic deformation plays a very special role at a temperature of 150 °C (see panel (a) in Figure 10). At this temperature, the fracture curve, after its elastic region, passes into the plastic deformation region, skipping the stage of martensitic transformation (Figure 10, panel (a)). The deformation of the alloy in this state is provided mainly by plastic shear, whose maximum value before failure is as high as 36%.

Thus, analysis of the physical and mechanical properties of the sample showed that the obtained TiNi wire has suitable parameters to be used as an implant material providing a double-barreled enterostomy. Its MT temperatures, the value of martensitic transformation stress, and deformation behavior make the material appropriate for use in the human body. Importantly, its shape-memory effect manifests when the material is heated to the human body temperature.

### 3.4. Application of TiNi-Based Material for Mikulicz Double-Barreled Enterostomy

**Figure 11:**
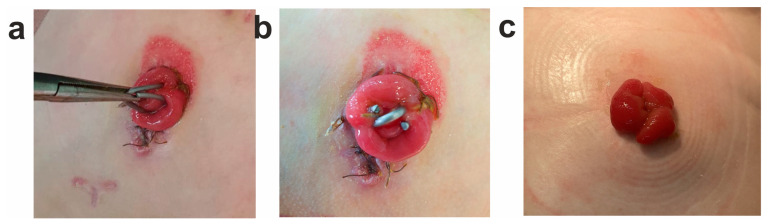
Photos of double-barrel enterostomy with compression anastomosis in a newborn child with meconium ilex: (**a**) application of TiNi shape-memory clip to the interstomal spur; (**b**) clip on a double-barreled enterostomy; (**c**) double-barreled enterostomy after removal of the clip and formation of an inter-intestinal compression anastomosis.

In total, the study involved 46 children aged from 1 day to 6 months who had double-barreled enterostomas formed during surgical treatment of congenital or acquired intestinal diseases. The experimental group included 18 children who underwent compression anastomosis in the location of the interstoma spur of the enterostomy using a shape-memory clip made of TiNi (see an example in Figure 11). The remaining 28 children from the control group did not undergo compression anastomosis.

The total duration of the ostomy period observed in the control group was shorter than in the experimental group (*p* = 0.032, U = 55). This is explained by the fact that early closure of the enterostomy occurred in some patients without the use of a clip, and some other patients died before the reconstructive stage. The condition of children in the experimental group was observed to stabilize after the formation of a compression anastomosis, which allowed them to be discharged from the hospital for a “medical break” and to close their stoma routinely. It should be noted that the mortality that prevailed in the control group (*p* = 0.001, F = 11.100) was due to the more severe course of underlying diseases (prematurity, bronchopulmonary dysplasia, or intraventricular hemorrhage) and was probably not associated with the absence of compression anastomosis.

There were no statistically significant differences in the incidence of enterostomy complications between the studied groups (*p* > 0.05). However, for the children of the control group, enterostomy reconstruction was more often required due to increased losses of intestinal chyme and persistent obstruction of the efferent colon (*p* = 0.015, F = 6.400) (see Table 5). Pathological loss of chyme, which was an indication for the application of a compression clip in the experimental group, was practically absent after the formation of anastomosis and restoration of passage through the intestine. In the experimental group, enterostomy complications that required surgical treatment under general anesthesia (CDC IIIB) were less common than in the control group (*p* = 0.003, F = 9.800), and they less often required additional drug therapy (CDC II) in the form of prolonged antibiotic therapy or topical application of healing ointments (*p* = 0.022, F = 6.200).

Thus, we conclude that the use of TiNi shape-memory compression clip in children with double-barreled enterostomies improves the results of surgical treatment, as it permits reducing pathological losses of intestinal chyme and restoring patency in the distal intestines. As a result, the need for reconstruction of the stoma and, accordingly, repeated surgery and general anesthesia are eliminated.

## 4. Conclusions

In this work, we report on the preparation of TiNi-alloy-based wire with the shape-memory effect, which was then used for two-brunch compression clips for surgery. The composition and structure of the TiNi material, as well as its martensitic and physical–chemical properties, were also studied. The TiNi alloy was found to consist of B2 and B19′ phases with inclusions of secondary-phase particles of Ti_2_Ni, TiNi_3_ and Ti_3_Ni_4_. Its TiNi matrix was slightly enriched in Ni (50.3 at.% of Ni), and its Ti_3_Ni_4_ inclusions were observed to retain coherent conjugation with the matrix.

The obtained alloy material had a homogeneous grain structure with an average grain size of 19 ± 0.3 μm and with equal quantities of grain boundaries of special and general types. In addition, the wire drawn from such material had a surface oxide layer, whose presence should provide improved biocompatibility and promote the adhesion of protein molecules.

Two-stage MT was detected in the TiNi alloy employed in this work, with the intermediate R-phase that is formed easily because of fine Ti_3_Ni_4_ particles distributed in the TiNi matrix. Overall, the obtained TiNi wire was concluded to exhibit physical and mechanical properties suitable for its use as an implant material providing a double-barreled enterostomy. Importantly, its shape-memory effect manifests when the material is heated to the human body temperature. 

The produced TiNi wire was then used for manufacturing compression clips with the shape-memory effect that were then applied in surgery. The medical experiment that involved 46 children demonstrated that the use of such TiNi clips in children with double-barreled enterostomies permitted improved results of surgical treatment. Such clips were shown to help reduce pathological losses of intestinal chyme and restore patency in the distal intestines so that no additional reconstruction of the stoma and repeated surgery with general anesthesia was necessary after their use.

## Figures and Tables

**Table 1:** Phase composition of TiNi wire according to XRD data.

Phase	Content, vol.%	Lattice Parameters, Å
TiNi (B2)	71.9	a = 3.0144
TiNi (B19′)	6.3	a = 2.7826
		b = 4.6198
		c = 4.2130
Ti_2_Ni	12.0	a = 11.3190
TiNi_3_	4.3	a = 5.0988
		c = 8.3347
Ti_3_Ni_4_	5.5	a = 11.3137
		c = 5.0743

**Table 2:** Content of main elements in wire’s cross-sectional surface and subsurface layers according to EDX data.

Phase	Elemental Content *, at.%	
	Ti	Ni
TiNi (position 1)	49.7	50.3
Ti_2_Ni (position 2)	66.03	33.97
TiNi_3_ (position 3)	26.83	73.17

* Carbon and oxygen present in the material were excluded from the calculation because of low detection accuracy of EDX for light elements.

**Table 3:** Temperatures of MTs found for the TiNi wire from its temperature dependence of electrical resistivity.

Cycle	Temperature, °C				
	T_R_	Ms	Mf	As	Af
1	44 ± 2	24 ± 2	5 ± 2	32 ± 2	56 ± 2
2	38 ± 2	17 ± 2	−2 ± 2	32 ± 2	56 ± 2
10	25 ± 2	0 ± 2	−22 ± 2	33 ± 2	50 ± 2

**Table 4:** Maximum and minimum martensitic transformation stresses of the TiNi wire.

Temperature, °C	−196		0		25		150	
$\varepsilon_{f}$, %/$\sigma_{f}$, MPa	$\varepsilon_{f}$	$\sigma_{f}$	$\varepsilon_{f}$	$\sigma_{f}$	$\varepsilon_{f}$	$\sigma_{f}$	$\varepsilon_{f}$	$\sigma_{f}$
TiNi wire	21	972	44	974	49	1013	36	810

**Table 5:** Results in the form of indexes for experimental and control groups.

Index	Experimental Group (*n* = 18)	Control Group (*n* = 28)
Fatal cases	1 (6%)	15 (54%)
Stoma closure	16 (89%)	12 (43%)
No complications	3 (17%)	4 (14%)
Three or more complications	7 (39%)	9 (32%)
Peristomal dermatitis	10 (56%)	15 (54%)
Stoma prolapse	4 (22%)	9 (32%)
Bleeding	4 (22%)	6 (21%)
Hyperproduction of chyme	11 (61%)	11 (39%)
Liver failure	2 (11%)	6 (21%)
Stoma necrosis	0	5 (18%)
Parastomal eventration	2 (11%)	2 (7%)
Intestinal obstruction	2 (11%)	10 (36%)

## Data Availability

Not applicable.
